# Clinical complete response after nivolumab administered as a third-line treatment for unresectable advanced gastric cancer with peritoneal dissemination: A case report

**DOI:** 10.1016/j.ijscr.2021.106161

**Published:** 2021-06-30

**Authors:** Toshiaki Komo, Takahisa Suzuki, Hirofumi Tazawa, Haruki Sada, Hiroshi Morimoto, Norimitsu Shimada, Naoto Hadano, Takashi Onoe, Takeshi Sudo, Yosuke Shimizu, Hirotaka Tashiro

**Affiliations:** a Department of Surgery, National Hospital Organization, Kure Medical Center Chugoku Cancer Center, Japan; b Department of Gastroenterological and Transplant Surgery, Applied Life Sciences, Institute of Biomedical and Health Sciences, Hiroshima University, Japan

**Keywords:** AE, Adverse even, irAE, immune-related adverse events, PD, Programmed death, OS, Overall survival, PFS, Progression free survival, ORR, Overall response rate, CT, Computed tomography, CR, Complete response, PR, Partial response, Case report, Nivolumab, Complete response, Gastric cancer

## Abstract

**Introduction and importance:**

Nivolumab, which is a fully human IgG4 PD-1 immune checkpoint inhibitor antibody, has been recommended as a third-line treatment based on the results of the ATTRACTION-2 study involving patients with unresectable advanced gastric cancer.

**Case presentation:**

A 69 year-old woman was referred to our department with a diagnosis of gastric cancer based on an upper gastrointestinal endoscopy during a medical examination. The endoscopy, along with various tests, helped establish a diagnosis of unresectable advanced gastric cancer (cT4aN3aM1P1c, cStage IV) with peritoneal dissemination. The first and second-line chemotherapy administered was S-1 plus oxaliplatin followed by ramucirumab and nab-paclitaxel, respectively. In this case, the disease was evaluated as progressive disease due to increased peritoneal dissemination. Nivolumab was administered as the third-line treatment. The patient developed interstitial pneumonia after nine courses of nivolumab, for which chemotherapy was discontinued and prednisolone treatment was initiated. The patient had a complete response to treatment endoscopically, 9 months after the last administration of nivolumab. After that, there was no recurrence of the cancer, despite there being no treatment for 5 months.

**Clinical discussion:**

It was suggested that the therapeutic effect of nivolumab could be maintained for a long period after discontinuation of its administration. In addition, a correlation has been reported between the treatment efficacy and immune-related adverse events associated with nivolumab.

**Conclusions:**

The synergistic effect of the sustained effect of nivolumab and later-line treatment may contribute to the prolongation of survival after discontinuation of nivolumab in patients who are refractory or intolerant to treatment.

## Highlights

• Nivolumab is recommended as third-line treatment in patients with unresectable advanced gastric cancer.
• Nivolumab is expected to have a long tail effect.
• The synergistic effect of nivolumab and the later-line treatment may contribute to the prolongation of survival.

## 1 Introduction

The effectiveness of cancer immunotherapy using immune checkpoint inhibitors has been proven in recent years [1].

Nivolumab, which is a fully human Immunoglobulin (Ig) G4 monoclonal antibody targeting programmed death (PD)-1, was evaluated in phase 3 of the ATTRACTION-2 study in patients with unresectable advanced, recurrent gastric, or gastroesophageal junction cancer treated with ≥2 prior chemotherapy regimens [2]. The results of the 2-year follow-up of the ATTRACTION-2 study demonstrated that although the overall response rate (ORR) was only 11.9%, the 2-year overall survival (OS) rate of the response cases was 61.3%, showing a sustained therapeutic effect [3].

Based on the results of this study, nivolumab is expected to have a long-tail effect. Furthermore, it was reported that this effect continued for more than 20 weeks after discontinuation of nivolumab administration in patients with lung cancer [4].

In addition, grade 3 or 4 adverse events (AEs) reportedly occur with a probability of 11.8% when nivolumab is administered [3], and it is especially important to deal with immune-related AEs (irAEs). However, a correlation has been reported between nivolumab efficacy and irAEs [5].

Herein, we report a case of interstitial pneumonia caused by nivolumab administered as a third-line treatment for unresectable advanced gastric cancer with peritoneal dissemination, which achieved a clinical complete response even without treatment follow-up.

The present work has been reported in accordance with the SCARE criteria [6].

## 2 Case presentation

**Fig. 1:**
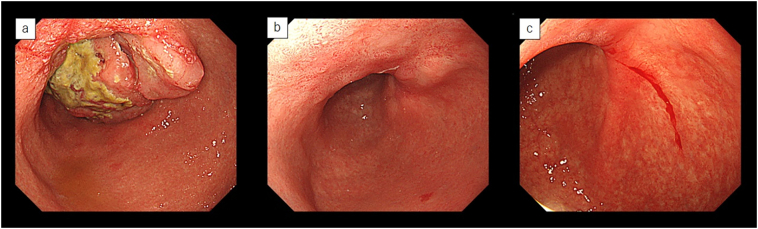
Upper gastrointestinal examination revealed a type 3 lesion in the lesser curvature of the stomach, in the posterior wall of the body of the stomach (a). The primary lesion had reduced in size and ulceration had disappeared (b). The primary lesion disappeared and was observed as comparatively gentle mucosa (c).

**Fig. 2:**
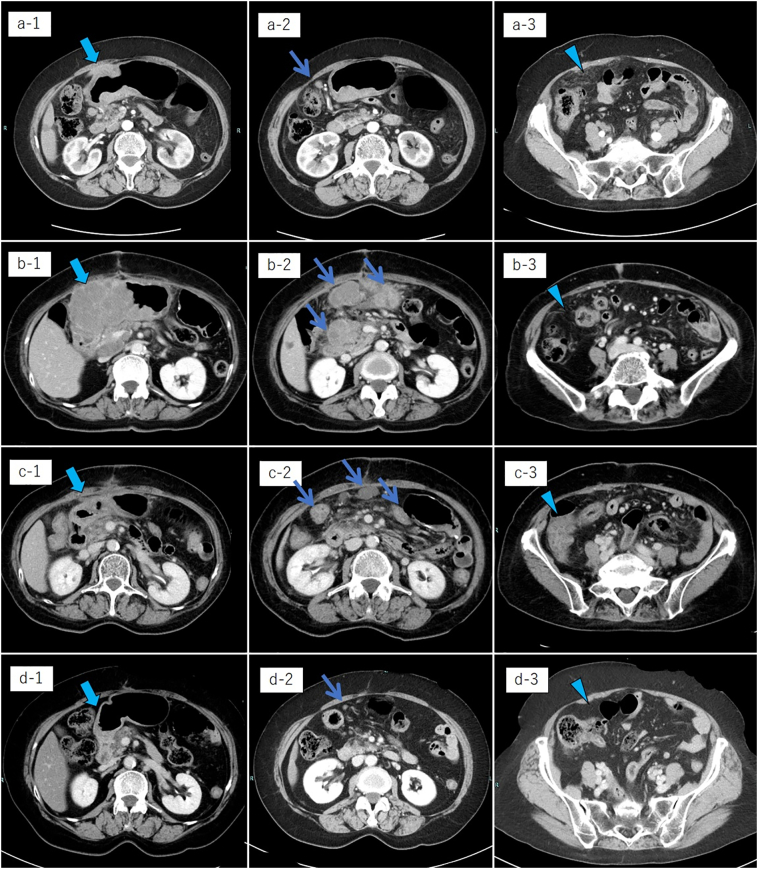
Contrast-enhanced CT examination showed the transition of the therapeutic effect in the primary lesion in the body of the stomach (thick arrow), lymph nodes (thin arrow), and peritoneal dissemination (arrow head).

**Fig. 3:**
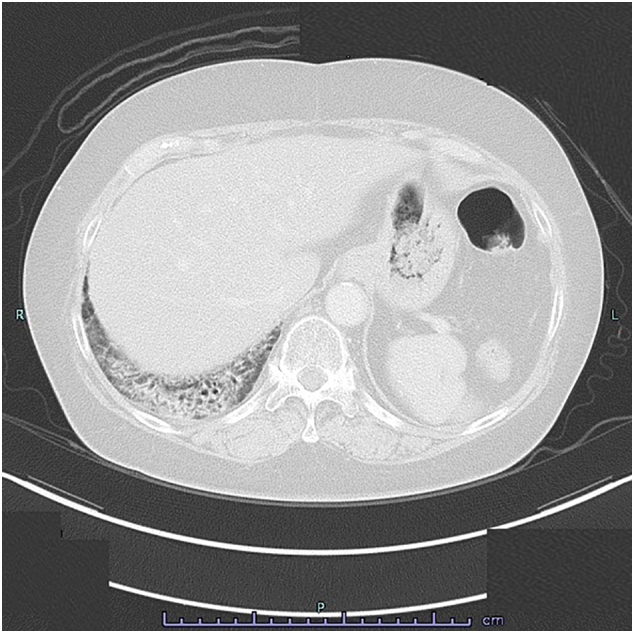
Contrast-enhanced CT examination revealed interstitial pneumonia developed after nine courses of nivolumab.

**Fig. 4:**
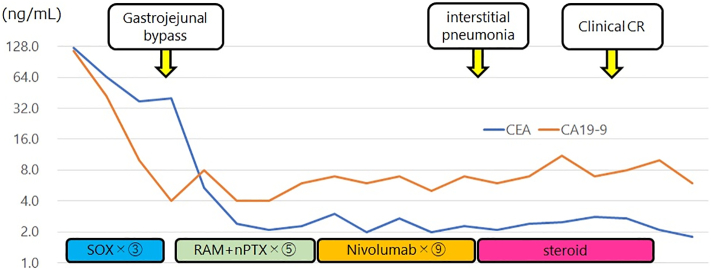
The course of treatment and the transition of tumor markers.

A 69 year-old woman was referred to our department because of a diagnosis of gastric cancer established using upper gastrointestinal endoscopy during a medical examination. The upper gastrointestinal examination revealed a type 3 lesion in the lesser curvature of the stomach, in the posterior wall of the body of the stomach (Fig. 1a). Biopsy revealed a poorly differentiated tubular adenocarcinoma. Contrast-enhanced computed tomography (CT) revealed irregular wall thickening with a contrast-enhancing effect on the body of the stomach, and lymphadenopathy occurred frequently (Fig. 2a-1, 2). In addition, an irregular increase in concentration was observed in the omentum, and peritoneal dissemination was suspected (Fig. 2a-3). As a result of various tests, the patient was diagnosed with unresectable advanced gastric cancer (cT4aN3aM1P1c, cStage IV) with peritoneal dissemination. Chemotherapy was administered with S-1 plus oxaliplatin as the first-line treatment. The patients developed a passage obstruction after the administration of three courses of S-1 plus oxaliplatin; therefore, a gastrojejunal bypass surgery was performed. Contrast-enhanced CT examination revealed increased primary lesions and lymph nodes, reducing the irregular spread of the omentum (Fig. 2b-1, 2, 3). A combination of ramucirumab and nab-paclitaxel was used for the second-line chemotherapy. A subsequent upper gastrointestinal examination revealed that the primary lesion in the gastric body had shrunk and ulceration had disappeared after five courses of ramucirumab plus nab-paclitaxel (Fig. 1b). This was classified endoscopically as a partial response (PR). Contrast-enhanced computed tomography (CT) revealed decreased primary lesions and lymph nodes (Fig. 2c-1, 2). The patient's disease was classified as progressive according to the Response Evaluation Criteria in Solid Tumors (RECIST) version 1.1, due to increased peritoneal dissemination (Fig. 2c-3). Consequently, nivolumab (240 mg every 2 weeks) was administered as the third-line chemotherapy. The patient developed interstitial pneumonia after nine courses of nivolumab (Fig. 3). Chemotherapy was discontinued, and prednisolone treatment was initiated for interstitial pneumonia. Subsequently, the primary lesion in the body of the stomach disappeared with a comparatively gentle mucosa observed in its place (Fig. 1c). This was classified endoscopically as a complete response (CR), 9 months after the last administration of nivolumab, despite being followed-up. Contrast-enhanced CT showed that wall thickening of the primary lesion had disappeared, with the involved lymph nodes and peritoneal dissemination having reduced in size (Fig. 2d-1, 2, 3). At the same time, the interstitial pneumonia was cured, and prednisolone treatment was terminated. There was no recurrence of the tumor, despite the patient being under no treatment for 5 months. The course of treatment and transition of the tumor markers are shown in Fig. 4.

## 3 Discussion

Recent developments in systemic chemotherapy have improved the prognosis of patients with unresectable advanced or recurrent gastric cancer, with the median survival time (MST) prolonged to approximately 15 months [7], [8].

In Japan, nivolumab monotherapy has been recommended as a third-line treatment after the results of the ATTRACTION-2 study demonstrated its efficacy [9]. The results of the 2-year follow-up of the ATTRACTION-2 study demonstrated that the ORR was greater in the nivolumab group than in the placebo group. Although the ORR was only 11.9%, the 2-year OS rate was 61.3% in patients in the nivolumab group whose treatment responses were classified as CR or PR. Only three patients (1.1%) had CR. The number of patients with CR increased from zero to three during the 2-year follow-up [3]. This indicates the sustained therapeutic effect of nivolumab.

Thus, nivolumab is expected to have a long tail effect on the Kaplan-Meier survival curve [10]. Furthermore, this effect may continue after the discontinuation of nivolumab administration in patients with lung cancer [11]. Osa et al. [4] reported that nivolumab binding on the memory T cells in the blood is detectable more than 20 weeks after the final dose, regardless of the number of nivolumab doses and the content of subsequent treatment. Pharmacokinetically, it was suggested that the therapeutic effect of nivolumab could be maintained for a long period after its discontinuation. Kato et al. [12] retrospectively evaluated patients with unresectable advanced or recurrent gastric cancer who underwent cytotoxic chemotherapy. These patients received cytotoxic chemotherapy after the progression of immune checkpoint inhibitors or as a third-line treatment without prior exposure to immune checkpoint inhibitors. The ORR was significantly higher in the former cases than in the latter (31% vs. 10%, respectively). It has been suggested that the synergistic effect of the sustained effect of nivolumab with the later-line treatments may contribute to a prolonged survival after discontinuation of nivolumab in patients who are refractory or intolerant to treatment.

Reportedly, grade 3 or 4 AEs occur with a probability of 11.8% when nivolumab is administered [3]. It is particularly important to deal with irAEs. Grade 3 or 4 interstitial pneumonia is reported to occur at a frequency of 0.3% when nivolumab is administered [3]. In such cases, nivolumab should generally be discontinued and prednisolone should be used for treatment [13]. Prednisolone has not been shown to affect the sustained therapeutic effect of nivolumab after its discontinuation [4]. Thus, even if subsequent treatment with cytotoxic chemotherapy is not possible, the sustained treatment effect of nivolumab can be expected during treatment for interstitial pneumonia. On the other hand, it was reported that non-small cell lung cancer patients with irAEs caused by nivolumab had significantly higher ORRs than such patients without irAEs. Similarly, the progression-free survival among patients with irAEs was longer than that among patients without irAEs [5]. It was suggested that there was a correlation between irAEs and treatment efficacy in patients treated with nivolumab.

In this case, the therapeutic effect of nivolumab was maintained because the state of complete binding of T lymphocytes persisted even after discontinuation of the drug. Moreover, this effect was not affected by the administration of steroids. During treatment with steroids, the sustained treatment effect of nivolumab lasted 36 weeks, which was well over the previously reported sustained effect duration of 20 weeks. If the sustained effect of nivolumab is shown to be effective, a synergistic effect in later-line treatments may be expected in the longer term. Although the mechanisms underlying the association of irAEs with outcomes of treatment with nivolumab are unknown, in addition to the sustained effect of nivolumab and its synergistic effect with the later-line treatment, the incidence of irAEs may have contributed to the prolongation of progression-free survival [14]. Thus, nivolumab may be an effective drug for first or second-line chemotherapy in such cases. The ATTRACTION-4 study has progressed to part 2 (phase III), where treatment with nivolumab plus cytotoxic chemotherapy is being compared with treatment using a placebo plus cytotoxic chemotherapy as a first-line therapy for unresectable advanced cancer [15]. If nivolumab plus chemotherapy is the first line of treatment, it is expected to have a sustained therapeutic effect, along with a synergistic effect with the subsequent lines of treatment with nivolumab. It is expected that OS will be further extended.

## 4 Conclusions

It has been suggested that the therapeutic effect of nivolumab can be maintained for a long time even after discontinuation of nivolumab administration, which is expected to have a long-term therapeutic effect as well as a synergistic effect with the subsequent line of treatment. In addition, the incidence of irAEs associated with nivolumab may have contributed to the prolongation of survival outcomes. Further studies are required to confirm the benefits of nivolumab therapy.

## Ethical approval

The study such as this case report was exempted from ethical approval by the Institutional Review Board of National Hospital Organization, Kure Medical Center, and Chugoku Cancer Center.

## Sources of funding

The authors declare that this study was not funded externally.

## Authors' contributions

TK drafted the manuscript. TS and HT reviewed and edited the manuscript. TK, TS, and HT participated in the care of the patients. HA, HM, NS, NH, TO, TS and YS participated in critical revision of the manuscript. All authors read and approved the final manuscript.

## Guarantor

Takahisa Suzuki

3-1 Aoyama-cho, Kure City, Hiroshima, 737-0023, Japan

Tel: +81-82-322-3111, FAX: +81-82-321-0478

E-mail: takahisa-suzuki@umin.ac.jp

## Research registration

There are no new surgical technique or new equipment/technology.

## Consent for publication

When obtaining informed consent for surgical procedures, general consent for publication and presentation was obtained from the patient.

## Patient perspective

The patient shared their perspective on the treatments they received.

## Disclosure of commercial interest

None of the authors has any commercial interest in the subject of this study or received any financial or material support for this study.

## Provenance and peer review

Not commissioned, externally peer-reviewed.

## Declaration of competing interest

The authors declare that they have no conflicts of interest.
